# *PDGFRA*, *KIT*, and *KDR* Gene Amplification in Glioblastoma: Heterogeneity and Clinical Significance

**DOI:** 10.1007/s12017-023-08749-y

**Published:** 2023-08-23

**Authors:** Bianca Soares Carlotto, Patricia Trevisan, Valentina Oliveira Provenzi, Fabiano Pasqualotto Soares, Rafael Fabiano Machado Rosa, Marileila Varella-Garcia, Paulo Ricardo Gazzola Zen

**Affiliations:** 1 Graduate Program in Pathology, Universidade Federal de Ciências da Saúde de Porto Alegre (UFCSPA), Porto Alegre, RS Brazil; 2 Colorado Genetics Laboratory, Department of Pathology, School of Medicine, University of Colorado, Aurora, CO USA; 3 Pathology Section, Grupo Hospitalar Conceição (GHC), Porto Alegre, RS Brazil; 4 Regenerar - Centro de Medicina da Dor, Porto Alegre, RS Brazil; 5 Department of Internal Medicine, Clinical Genetics, Universidade Federal de Ciências da Saúde de Porto Alegre (UFCSPA), Porto Alegre, RS Brazil; 6 Irmandade da Santa Casa de Misericórdia de Porto Alegre (ISCMPA), Porto Alegre, RS Brazil; 7 Department of Medicine, Medical Oncology Division, School of Medicine, University of Colorado Anschutz Medical Campus, Aurora, CO 80045 USA

**Keywords:** Glioblastoma, *PDGFRA*, *KIT*, *KDR*, Genetic heterogeneity, FISH

## Abstract

Glioblastoma (GBM) is the most frequent tumor of the central nervous system, and its heterogeneity is a challenge in treatment. This study examined tumoral heterogeneity involving *PDGFRA*, *KIT*, and *KDR* gene amplification (GA) in 4q12 and its association with clinical parameters. Specimens from 22 GBM cases with GA for the 4q12 amplicon detected by FISH were investigated for homogeneous or heterogeneous coamplification patterns, diffuse or focal distribution of cells harboring GA throughout tumor sections, and pattern of clustering of fluorescence signals. Sixteen cases had homogenously amplification for all three genes (45.5%), for *PDGFRA* and *KDR* (22.7%), or only for *PDGFRA* (4.6%); six cases had heterogeneous GA patterns, with subpopulations including GA for all three genes and for two genes - *PDGFRA* and *KDR* (13.6%), or GA for all three and for only one gene - *PDGFRA* (9.1%) or *KIT* (4.6%). In 6 tumors (27.3%), GA was observed in focal tumor areas, while in the remaining 16 tumors (72.7%) it was diffusely distributed throughout the pathological specimen. Amplification was universally expressed as double minutes and homogenously stained regions. Coamplification of all three genes *PDGFRA*, *KIT*, and *KDR*, age ≥ 60 years, and total tumor resection were statistically associated with poor prognosis. FISH proved effective for detailed interpretation of molecular heterogeneity. The study uncovered an even more diverse range of amplification patterns involving the 4q12 oncogenes in GBM than previously described, thus highlighting a complex tumoral heterogeneity to be considered when devising more effective therapies.

**Supplementary Information:**

The online version contains supplementary material available at 10.1007/s12017-023-08749-y.

## Introduction

Glioblastoma (GBM) is the most common primary malignant lesion in the brain and other central nervous system (CNS) organs (14.3% of all tumors and 49.1% of malignant tumors); it represents most of gliomas (58.4%) and associates with median survival of 8 months (Ostrom et al., 2021). GBM incidence is higher in older and in male adults; female sex and older age (40+ years) were associated with poorer survival (Ostrom et al., 2020, 2021). The average annual age-adjusted incidence rate of malignant brain and other CNS tumors was 7.06 per 100,000 between 2014 and 2018 in the US (Ostrom et al., 2021). In Southern Brazil (state of Rio Grande do Sul), incidence of CNS tumors in 2020 was estimated at 9.05 and 7.58 per 100,000, respectively in male and female adults (Instituto Nacional de Câncer José Alencar Gomes da Silva, 2019).

GBMs are highly heterogeneous tumors exhibiting regional and cellular genotypic and phenotypic variations (Park et al., 1995). Tumor heterogeneity is one of the significant barriers to the development of effective therapeutic approaches in solid tumors (Hanahan & Weinberg, 2011) and this high-grade glioma is well known by therapeutic resistance and aggressiveness (Cantanhede & de Oliveira, 2017). The GBM heterogeneity not only manifests as a genetic and phenotypic variation in different individuals (intertumoral heterogeneity), but also as a simultaneous display of subclonal diversity (intermingled or spatially separated) within a tumor (intratumoral heterogeneity - IH) (Burrell et al., 2013). Heterogeneity is expressed as different gene, molecular and cellular features leading to lesions with distinct genetic, molecular, and morphological profiles, transcription and expression patterns, configurations of vascular proliferation, metabolism, micro-environment, and metastatic potential; all factors playing a key role in tumor progression and therapeutic resistance (Belsuzarri et al., 2018; Hanahan & Weinberg, 2011; Little et al., 2012; Snuderl et al., 2011). The striking IH originates from the combination of regional genetic variation and cellular hierarchy, frequently controlled by distinct groups of cancer stem cells (Schonberg et al., 2014).

Genomic and in situ fluorescent (FISH) and chromogenic hybridization (CISH) studies in GBM have detected a profound IH in the amplification patterns of receptor tyrosine kinases (RTK) and drug target genes, such as *EGFR*, *MET*, and *PDGFRA* (Burford et al., 2013; Little et al., 2012; Snuderl et al., 2011; Szerlip et al., 2012). *PDGFRA* maps at 4q12 and is contiguous to *KIT* and *KDR*, two other RTK and drug target genes (Burford et al., 2013). *PDGFRA*, *KIT*, and *KDR* apparently developed from a common ancestral gene and frequently coamplify in GBM (Joensuu et al., 2005).

We used FISH to examine rearrangements in *ROS1* and *NTRK1*, amplification of *PDGFRA*, *KIT*, and *KDR*, and deletion of *RB1*, and to verify their potential clinical significance in a Brazilian cohort of adult GBM patients (Trevisan et al., 2019). *RB1* was deleted in 16% of cases and *PDGFRA* was amplified in 20%, often coamplified with *KDR* (> 90%) and *KIT* (> 60%). Complications after surgery, older age and right-sided tumors were independent variables associated with patient survival.

Subsequently, we re-investigated in detail the patterns of gene amplification (GA) of *PDGFRA*, *KIT*, and *KDR* in those GBM specimens carrying GA, and its association with *RB1* deletion and other clinical variables. These data are presented here.

## Materials and Methods

### Datasets and FISH

From 113 cases classified as GBM NOS (Trevisan et al., 2019), according to OMS 2016 guidelines (Ohgaki et al., 2016), a retrospective cohort of 22 cases with GA involving *PDGFRA*, *KIT*, and *KDR* was formed. GA was accepted when the gene:control copy number ratio was ≥ 2, or there were ≥ 15 copies of gene in ≥ 10% of tumor cells (Cappuzzo et al., 2005). All formalin-fixed paraffin-embedded (FFPE) tumor sections were from surgical resections. H&E-stained slides were centrally revised and representative tumoral sections demarcated by a pathologist (VOP). Gene copy numbers were investigated by 4-color (*PDGFRA*/*KIT*/*KDR*/CEP4) and 2-color FISH (*RB1*/LSI 13q34) in sequential 4-μm FFPE tissue sections. Detailed assay protocols using Abbott Molecular DNA probes (*KIT* labeled in red, CEP4 labeled in aqua, *RB1* labeled in red, and LSI 13q34 labeled in green) and homebrew DNA probes (*PDGFRA* labeled in green and *KDR* labeled in gold) were described (Trevisan et al., 2019).

The defined tumor areas were entirely scanned using objective 40 × and dual red/green or yellow/aqua filters to determine whether the GA pattern was evenly or focally distributed. At least 100 tumor nuclei in 8 tumor regions were scored. Nuclei selection was based on factors such as average or larger size to minimize the effect of truncation, presence of intact chromatin, and no detectable overlapping. FISH signals were individually enumerated as precisely as possible, using a fluorescence microscope with Z-stacking and 100 × objective, carefully scanning the nuclei content on their entire depth. It was also annotated if the fluorescent spots representing copies of a given gene were tightly or loosely clustered within each nucleus. *RB1* deletion was considered when ratio gene:control RB1:13q34 < 0.8 (Rodriguez et al., 2008) or there was a single copy of gene signal in > 50% of tumor nuclei (Goldhoff et al., 2012).

### Statistical Analysis

The quantitative variables were described as mean (and standard deviation) or median (and interquartile range). Absolute and relative frequencies were used for categorical variables, as well as Chi-square test and Fisher exact test. To evaluate survival, Kaplan–Meier method was applied, and the curves were compared using the log-rank test. To control confounding factors, multivariate Cox Proportional Hazards analysis was used. The criterion for entering the variable in the multivariate model was that it had a *p *value < 0.10 in the bivariate analysis. The significance level adopted was 5% (*p* < 0.05) and the analyses were performed in the SPSS program version 21.0.

## Results

Twenty-two patients were included in this study, with their clinical data summed up in Table 1 and presented in detail in Supplementary Table 1. Most subjects were female (59.1%) and ranged in age at diagnosis from 23 to 71 years (mean 53.9; SD 11.6; 95.4% were ≥ 40 years). Focal disturbance (59.1%) and increased intracranial pressure (50%) were the main symptoms at diagnosis. Inpatient interval ranged from 9 to 167 days (median 18.5 days) and median overall survival (OS) was 7.2 months (239.5 days). Partial resections were performed in 77.3% of patients, and the most common additional treatment was radiotherapy (RT) (68.2%), although in 27.3% of cases it was not possible to confirm that the treatment was performed.

**Table 1:** Clinical data of 22 patients

Variables	*n* (%)
Age (years) - mean ± SD	53.9 ± 11.6
Age	
< 40 years	1 (4.5)
40–59 years	12 (54.5)
≥ 60 years	9 (40.9)
Sex	
Female	13 (59.1)
Male	9 (40.9)
Clinical features	
Focal disturbance	13 (59.1)
Increase ICP	11 (50.0)
Behavioral change	3 (13.6)
Seizure	3 (13.6)
Decreased consciousness	1 (4.5)
GBM location	
Frontal	7 (31.8)
Temporal	4 (18.2)
Parietal	1 (4.5)
Other	10 (45.5)
Number of locations involved	
One region	12 (54.5)
2 or more regions	10 (45.5)
Side	
Right	13 (59.1)
Left	9 (40.9)
Prior CNS tumor	
No	19 (86.4)
Yes	2 (9.1)
Unknown	1 (4.5)
Resection	
Partial	17 (77.3)
Gross total	5 (22.7)
Post-surgical complications	
No	19 (86.4)
Yes	3 (13.6)*
Additional treatment	
Radiotherapy completed	9 (40.9)
Radiotherapy indicated not verified	6 (27.3)
Chemotherapy completed	2 (9.1)
Chemotherapy indicated not verified	1 (4.5)
None	1 (4.5)
Unknown	6 (27.3)

*ICP* intracranial pressure

*Hemorrhage, meningitis, and visual loss

### Analysis of *PDGFRA*, *KIT*, and *KDR* Gene Amplification

Among the 22 GBM cases harboring GA for any of the three genes evaluated, all had high level amplification (Supplementary Table 2). We investigated molecular heterogeneity in this GBM sample focusing on three aspects: how the coamplification of genes occurred in each cell, how the cells harboring GA were distributed spatially within the tumor section, and how the fluorescent signals indicating the presence of multiple copies of each gene were organized in each cell.

The phenomenon was homogeneously distributed within the tumor sections in 16 cases (72.7%) and displayed heterogeneity represented by foci of specific patterns in 6 cases (27.3%), as shown in Table 2. Ten of the cases with homogeneous amplification displayed GA for all 3 genes, while 5 displayed GA for 2 genes, *PDGFRA* and *KDR*, and one case displayed GA for only *PDGFRA*. Interestingly, the 6 cases with heterogeneous patterns showed areas with GA for the 3 genes concomitantly with areas with GA for only one (*PDGFRA* = 2 cases; *KIT* = 1 case) or 2 genes (*PDGFRA* and *KDR* = 3 cases). Therefore, among the 22 cases, six different amplification patterns were detected, three among the homogeneous cases—*PDGFRA*/*KIT*/*KDR* (45.5%), *PDGFRA*/*KDR* (22.7%), *PDGFRA* (4.6%), and three among the heterogeneous cases (*PDGFRA*/*KIT*/*KDR* + *PDGFRA*/*KDR* (13.6%), *PDGFRA*/*KIT*/*KDR* + *PDGFRA* (9.1%), and *PDGFRA*/*KIT*/*KDR* + *KIT* (4.6%). Overall, 16 specimens showed GA for the 3 genes (72.8%), 5 for 2 genes *PDGFRA* and *KDR* (22.7%), and one for only *PDGFRA* (4.6%). Of note, *PDGFRA* and *KDR* are not contiguous in the 4q12 amplicon, and the intermediate gene *KIT* was also found as singularly amplified in a subpopulation of one tumor.

**Table 2:** Presentation of gene amplification (GA) for genes in the 4q12 amplicon in adult GBM

Pattern of Gene Amplification Throughout Tumor Section	Distribution of Cells Harboring GA Throughout Tumor Section	Case ID in Trevisan et al. (2019)	Area	Gene		
				*PDGFRA*	*KIT*	*KDR*
Homogeneous	Diffuse	12		*	*	*
		17		*	*	*
		34		*	*	*
		64		*	*	*
		70		*	*	*
		71		*		*
		74		*	*	*
		85		*		
		87		*	*	*
		99		*	*	*
		105		*	*	*
		106		*	*	*
	Focal	9		*		*
		33		*		*
		68		*		*
		73		*		*
Heterogeneous	Diffuse	41	A	*	*	*
			B		*	
		58	A	*	*	*
			B	*		*
		62	A	*	*	*
			B	*		
		90	A	*	*	*
			B	*		
	Focal	2	A	*	*	*
			B	*		*
		69	A	*	*	*
			B	*		*

**Fig. 1:**
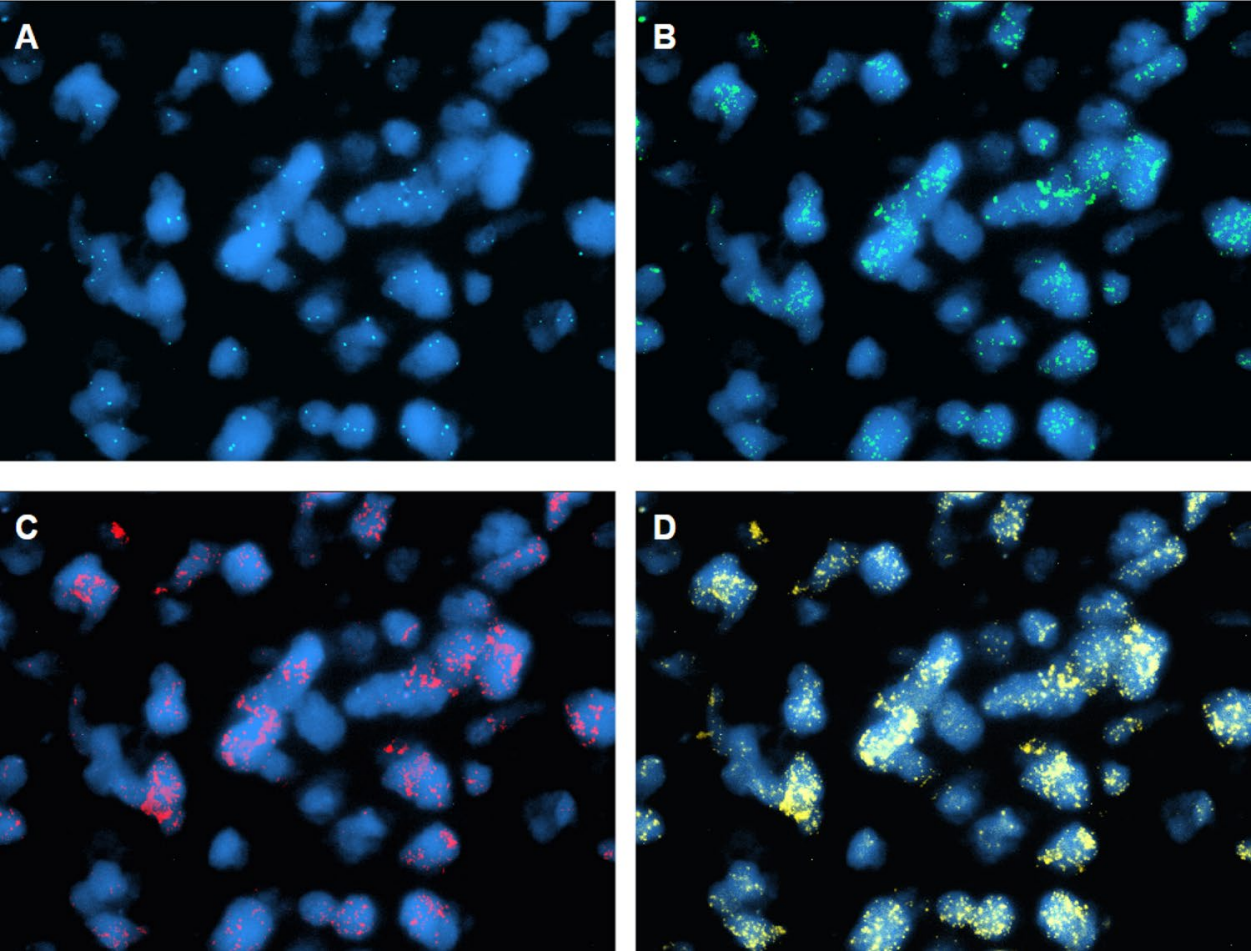
Case 12 - Segmented images of a microscope field (100x) showing coamplification of the three investigated genes, all presented as DM and HSR. **A** CEP4 (aqua signals) corresponded to a control probe recognizing centromere sequences of chromosome 4. **B** Copies of *PDGFRA* shown as green signals. **C** Copies of *KIT* shown as red signals. **D** Copies of *KDR* shown as yellow signals

In 16 cases, tumor nuclei carrying GA were found diffusely distributed within the section. In 6 cases, including tumors with homogeneous (cases 09, 33, 68, 73) and heterogenous patterns (cases 02 and 69), nuclei with GA appeared in focal, discrete tumor areas rather than in the entire scanned tumor. In all 22 cases and for all 3 genes, GA was exhibited not only as extrachromosomal double minutes (DM), with cosegregation of 2 or 3 genes, but also as homogeneously staining chromosomal regions (HSR), as shown in Fig. 1.

These GBM cases were additionally examined for *RB1* copy number loss and three cases (02, 41, and 62), which were positive for *PDGFRA*, *KIT*, and *KDR* GA, also harbored *RB1* deletion. In case 02, the large tumor area displaying GA for the three tested genes also displayed *RB1* loss. In the focal area positive for only *PDGFRA* and *KDR* GA, there was no *RB1* loss. In the other cases, the entire tumor section had *RB1* loss, despite focal areas exhibiting GA for the 3 genes and other discrete areas exhibiting GA for only *KIT* in case 41 and *PDGFRA* in case 62.

### Association with Survival

**Fig. 2:**
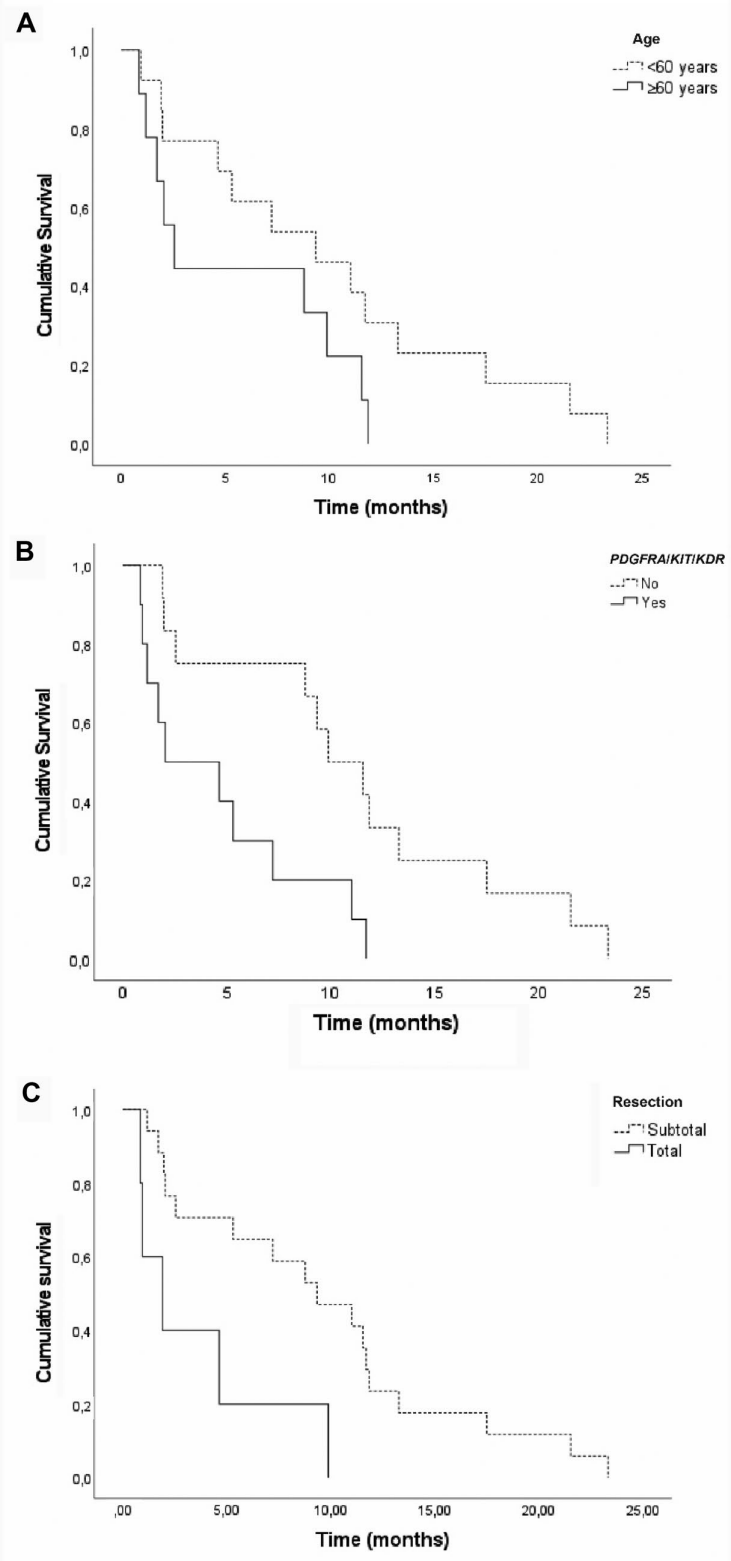
Cumulative survival estimated by the Kaplan–Meier method. **A** According to age group (HR = 13.9; 95% CI 2.82–68.3: *p* = 0.001). **B** According to the presence of homogeneous coamplification of the *PDGFRA/KIT/KDR* genes (HR = 10.5; 95% CI 1.24–89.5: *p* = 0.031). **C** According to resection type (HR = 9.30; 95% CI 1.62–53.3: *p* = 0.012)

We investigated the patient characteristics associated with poor prognosis in this sample and, after adjusting for confounding factors, homogeneous GA for all 3 genes—*PDGFRA*, *KIT*, and *KDR*, age ≥ 60 years, and total resection (Fig. 2) remained statistically associated with poor survival. Patients with homogeneous coamplification of *PDGFRA*, *KIT*, and *KDR* genes had 10.5 times higher risk of death than those with other amplification patterns. In addition, patients aged ≥ 60 years had 13.9 times higher risk of death than those aged under 60 years, and patients with total resection had 9.3 times higher risk of death than those with subtotal resection (Table 3 and Supplementary Table 3).

**Table 3:** Multivariate Cox Regression to assess predictors independently associated with death

Variables	HR (95% CI)	p
Age		
< 60 years	1.00	
≥ 60 years	13.9 (2.82–68.3)	**0.001**
*PDGFRA/KIT/KDR*		
No	1.00	
Yes	10.5 (1.24–89.5)	**0.031**
*PDGFRA/KDR*		
No	1.00	
Yes	0.33 (0.00–30.6)	0.630
*PDGFRA* + *PDGFRA/KIT/KDR*		
No	1.00	
Yes	3.80 (0.15–94.8)	0.416
Post-surgical complications		
No	1.00	
Yes	1.01 (0.19–5.43)	0.994
Resection		
Partial	1.00	
Gross total	9.30 (1.62–53.3)	**0.012**
Distribution of cells harboring GA Throughout tumor section		
Diffuse	1.99 (0.15–26.4)	0.602
Focal	1.00	
Amplification of 3 genes		
Yes	14.2 (0.47–428)	0.126
No	1.00	
Heterogeneity		0.309
No	1.00	
Sim	5.85 (0.20–175)	0.309

The significance of bold level adopted was 5% (p <0.05)

^*HR* Hazard Ratio; *95% CI* 95% confidence interval^

## Discussion

This study uncovered the coexistence of a diverse range of amplification patterns involving the RTK genes mapped in the 4q12 amplicon in adult GBM, thus suggesting a complex structure and dynamics of oncogene amplifications. We identified 16 cases with a single GA pattern (homogenous, 72.7%) and 6 cases with multiple GA patterns (heterogeneous, 27.3%). Focal amplifications were observed in 6 cases (27.3%), encompassing both homogeneous and heterogeneous cases and showing distinct GA patterns.

The 4q12 amplicon, including *PDGFRA*, *KIT*, and *KDR,* was previously examined in GBM by FISH (Burford et al., 2013; Joensuu et al., 2005), and TCGA genomic analyses (Snuderl et al., 2011). These studies detected similar patterns of amplification including all three genes (in 58.5%, 78.1%, and 42.9% of specimens, respectively for each listed study), two of the genes - *PDGFRA* and *KIT* (in 19.5%, 25.0%, and 23.8% respectively) and only *PDGFRA* (in 21.9%, 28.1%, and 33.3%, respectively). Amplification of *KDR* or *KIT* alone was never observed in these studies, nor by Szerlip et al. (2012) also using TCGA data. Our results differed since we found coamplification of *PDGFRA* and *KDR* (without *KIT*), as well as amplification of *KIT* alone in subpopulation of heterogeneous case. Concordantly, single amplification for *KIT* within the 4q12 amplicon was detected by CISH or FISH in 47% (15 out of 32) lower-grade gliomas harboring GA for only one of these 3 genes (Puputti et al., 2006).

The numerical discordance among the results may be due to the small number of specimens comprehensively investigated in the studies (*N* = 22 in ours, 21 in Snuderl et al., 2011, 32 in Joensuu et al., 2005, and 41 in Burford et al., 2013). Moreover, the different methodologies applied in the studies, in situ or genome-wide based assays, may have impacted the results. The genome-wide assays are very efficacious at detecting high degree of amplification in pure components of neoplastic cells, while the former, mainly FISH, offers a high-resolution platform for precise investigation of single tumor cells even when diluted out by non-amplified or contaminant non-tumor cells (Snuderl et al., 2011).

Remarkable heterogeneity in amplification of the RTK genes *EGFR*, *MET*, and *PDGFRA* in GBM has been reported in genomic and in situ studies (Little et al., 2012; Snuderl et al., 2011) and there is accumulating evidence that mosaicism of molecular patterns has profound implications for the design of effective chemo and targeted therapeutical regimens (Sottoriva et al., 2013). These studies described the presence of distinct intermingled or focal subpopulations of tumor cells and a frequent mutual exclusivity of GA for specific genes in individual GBM cells. Snuderl et al. (2011) additionally verified that the subclones shared numerous genetic mutations, thus supporting a common precursor for the distinct clones. We also compared the 4q12 GA with *RB1* loss and the 3 GBM cases displaying *RB1* loss were all heterogeneous for 4q12 GA. In one case, the subpopulation with 3-genes GA had *RB1* loss while the subpopulation with the 2-genes GA had not; in the other 2 cases, both the subpopulation with 3-genes and 1-gene GA had *RB1* loss. Despite the small number of specimens, it does not seem that mutual exclusivity was occurring.

Focal amplifications (FA) for *PDGFRA*-region in GBM were also previously reported (Snuderl et al., 2011; Sottoriva et al., 2013; Szerlip et al., 2012). FA are concerning events since they can be missed in in situ assays if not presented in the analytical sample, and in genome-wide assays if too diluted out to be detected. Moreover, FA can mediate targeted therapy resistance in cancer (Song et al., 2022).

Based on cytogenetic technology, the 4q12 amplicon in gliomas was known for a long time to be expressed in two forms: DM and intrachromosomal HSR (Muleris et al., 1994). DMs are autonomously replicating circular DNA of genomic origin, numerically unstable during mitotic cell division owing to unequal segregation between daughter cells (Shimizu, 2021). Conversely, HSRs are inserted into chromosomes and segregate equally to daughter cells, although the number of repetitive copies included in the amplicon may change during cell division. Both structures, DM and HSR, have been observed in our study for the 3 genes included in the 4q12 amplicon, as also described in GBM pathological specimens investigated by FISH or CISH (Little et al., 2012; Szerlip et al., 2012).

Nine of the 22 cases included in this study were investigated by Koshiyama et al. (2017) for aneuploidies of chromosomes 7 and 10, *EGFR* amplification, *PTEN*, *TP53* deletion, and 1p/19q deletions. All results for those patients are shown in Supplementary Table 4 but further analyses could not be done due to the small number of shared specimens between the studies.

In our study, the OS rate was 7.2 months (239.5 days), like data from the United States [median survival of 8 months and five-year relative survival of 6.8% (Ostrom et al., 2021)] and Germany [median OS of 9.5 and 13 months (Ening et al., 2015; Kaul et al., 2016)]. Median inpatient interval in our patients was 18.5 days (9–167), higher than in other studies [mean of 3 days (Fabrini et al., 2009), and median of 5 days (1–37) (Tully et al., 2016)]. In a recent study with Brazilian patients, treated with RT and concurrent and adjuvant temozolomide, median OS and progression-free survival times for the entire cohort were 17 and 9 months, respectively (Faustino et al., 2020).

Herein, GBM patients with *PDGFRA*, *KIT*, and *KDR* homogeneous coamplification had higher risk of death than those with other amplification patterns. Burford et al. (2013) also reported that GA for each of the 3 genes was significantly associated with poor survival, although patients with GA for two or three genes had worse clinical outcome than patients with only *PDGFRA* amplification, and patients with *PDGFRA* and *KIT* coamplification were younger at diagnosis and had better clinical evolution. Dono et al. (2021) evidenced that *RB1*-mutant *IDH*-WT GBM patients have improved progression-free survival and overall survival, and 4q12-amplified GBM *IDH*-WT patients have worse survival. In the search for associations between variables, a minimum power of 37% to a maximum of 67% was observed in study, indicating that further investigations in larger samples are recommended to strengthen our observations. Despite the suboptimal power, the study revealed relevant results on glioblastoma heterogeneity and will be useful in future meta-analysis.

This specific GBM subset harboring the 4q12 amplicon showed a similar association between poor prognosis and advanced age (> 60 years old) as the entire cohort investigated by Trevisan et al. (2019). Advanced age has been consistently associated with GBM poor prognosis (Cantanhede & de Oliveira, 2017; Ening et al., 2015; Koshiyama et al., 2017), likely due to the comorbidities and resistance to different treatments exhibited by elderly patients (Connon et al., 2016; Ohgaki et al., 2004). Older (> 65 years old) patients have shown higher expression levels of the PDGF family of genes than younger patients, highlighting the potential role of those genes as prognostic biomarkers (Cantanhede & de Oliveira, 2017).

Interestingly, this subset of GBM cases consisted mostly of female patients (59.1%), differently from the entire cohort (Trevisan et al., 2019) and from what is expected based on prevalence of GBM in male individuals. These are likely random results due to the small size of the patient subset. However, female sex has been associated with worse survival in GBM (Ostrom et al., 2021) and an individualized approach to patients management was recommended (Carrano et al., 2021).

Most patients in this subset were allocated to the focal disturbance group (59.1%) and, among these cases, almost all (92.3%) presented at least one type of motor deficit (Supplementary Table 1). The symptoms related with increased intracranial pressure were observed in 50.0% of cases, all presenting at least headache and/or intracranial hypertension (Table 1). The data agreed with different previous GBM studies; the common symptoms include focal neurological signs, seizures, mood, and personality changes, or symptoms of increased intracranial pressure (McKinnon et al., 2021; Preusser et al., 2011).

Statistically significant associations between greater extent of resection and longer survival were described on GBM and astrocytomas (Brown et al., 2016; Lacroix et al., 2001; McGirt et al., 2009; Stummer et al., 2008; Zinn et al., 2013). In our subset, patients with gross total resection had 9.3 times higher risk of death than those with subtotal resection, likely due to numerous confounding factors. The initial group of patients from which the current subset was selected (Trevisan et al., 2019), was seen in public hospitals sponsored by Brazilian federal health agencies. Typically, these patients had a late diagnosis, with functional performance already affected, and also delay in treatment (surgery, chemotherapy, and radiotherapy).

## Conclusion

Oncogene amplification is frequently associated with tumor progression and resistance to therapy, and spatial and temporal heterogeneity in the presentation of this phenomenon is considered a major challenge to improve patient survival. We herein evaluated comprehensively the patterns of *PDGFRA*, *KIT*, and *KDR* GA in a set of adult GBM from Southern Brazil and disclosed an expanded level of heterogeneity within the 4q12 amplicon. The FISH technique was confirmed as effective for identification and detailed interpretation of the heterogeneity in the pathological specimens. There was also an association of survival with the molecular profile (amplification of the 3 genes) and with clinical parameters (age and total resection). Although the analyses were done at just one time point, the initial resection, the high level of GA found in the subpopulations suggested a stable coexistence among them. In summary, our data uncovered a complex tumoral molecular heterogeneity that must be considered to devise more effective therapies to GBM.

### Supplementary Information

Below is the link to the electronic supplementary material.

Supplementary file1 (XLSX 276 KB)

## Data Availability

The datasets analyzed during the current study are available from the corresponding author on reasonable request.
